# Brown Adipose Tissue Activation by Cold Treatment Ameliorates Polycystic Ovary Syndrome in Rat

**DOI:** 10.3389/fendo.2021.744628

**Published:** 2021-10-14

**Authors:** Rongcai Ye, Chunlong Yan, Huiqiao Zhou, Yuanyuan Huang, Meng Dong, Hanlin Zhang, Xiaoxiao Jiang, Shouli Yuan, Li Chen, Rui Jiang, Ziyu Cheng, Kexin Zheng, Qiaoli Zhang, Wanzhu Jin

**Affiliations:** ^1^ Key Laboratory of Animal Ecology and Conservation Biology, Institute of Zoology, Chinese Academy of Sciences, Beijing, China; ^2^ University of the Chinese Academy of Sciences, Beijing, China; ^3^ College of Agriculture, Yanbian University, Yanji, China; ^4^ Institutes of Infectious Diseases, Beijing Ditan Hospital, Capital Medical University, Beijing, China; ^5^ Department of Human Reproductive Medicine, Beijing Obstetrics and Gynecology Hospital, Capital Medical University, Beijing Maternal and Child Health Care Hospital, Beijing, China

**Keywords:** rat, cold treatment, polycystic ovary syndrome, brown adipose tissue, ovary

## Abstract

Polycystic ovary syndrome (PCOS) is a common endocrine disease accompanied by energetic metabolic imbalance. Because the etiology of PCOS is complex and remains unclear, there is no effective and specific treatment for PCOS. It is often accompanied by various metabolic disorders such as obesity, insulin resistances, and others. Activated brown adipose tissue (BAT) consumes excess energy *via* thermogenesis, which has positive effects on energy metabolism. Our previous research and that of others indicates that BAT activity is decreased in PCOS patients, and exogenous BAT transplantation can improve PCOS rodents. Notably however, it is difficult to apply this therapeutic strategy in clinical practice. Therapeutic strategies of enhancing endogenous BAT activity and restoring whole-body endocrine homeostasis may be more meaningful for PCOS treatment. In the current study, the dehydroepiandrosterone-induced PCOS rat was exposed to low temperature for 20 days. The results show that cold treatment could reverse acyclicity of the estrous cycle and reduce circulating testosterone and luteinizing hormone in PCOS rats by activating endogenous BAT. It also significantly reduced the expression of steroidogenic enzymes as well as inflammatory factors in the ovaries of PCOS rats. Histological investigations revealed that cold treatment could significantly reduce ovary cystic follicles and increase corpus luteum, indicating that ovulation was recovered to a normal level. Concordant with these results, cold treatment also improved fertility in PCOS rats. Collectively, these findings suggest that cold treatment could be a novel therapeutic strategy for PCOS.

## Introduction

Polycystic ovary syndrome (PCOS) is one of the most common reproductive endocrine disorders affecting women of reproductive age (1, 2). Depending on the diagnostic criteria used, the incidence rate of PCOS is 9%–18% in women of reproductive age (3–5). PCOS is characterized by reproductive polycystic ovaries, hyperandrogenism and chronic anovulation (6–8). It is a heterogeneous and complex syndrome associated with dyslipidemia, obesity, insulin resistance, type 2 diabetes, and cardiovascular diseases (9–13). The etiology of PCOS is complex. A variety of environmental and genetic factors may lead to the occurrence of PCOS (14, 15).Thus, the treatment strategy of PCOS is not clear (16).

There is solid evidence of functional abnormalities of adipose tissue in PCOS patients, and it mainly manifests as insulin resistance and inflammation (17). Dysfunction of adipose tissue promotes the occurrence of metabolic disorders in peripheral tissues, and PCOS patients can exhibit larger adipocytes (18), lower lipoprotein lipolytic enzyme activity (18), and impaired catecholamine-mediated lipolysis capacity (19).

White adipose tissue (WAT) has the function of storing excess energy. In contrast with WAT, brown adipose tissue (BAT) dissipates energy as heat and consists of multiple small lipid droplets and numerous mitochondria (20). In human, BAT is mainly located in paravertebral, supraclavicular, perirenal, and cervical depots (21–23). High BAT activity is associated with protection against obesity and related metabolic alterations (24, 25). This association is attributed to the capacity of BAT to oxidate metabolites and generate heat (26). Using 18F-FDG positron emission tomography-computed tomography, we previously found that BAT activity was significantly decreased in a dehydroepiandrosterone (DHEA)-induced PCOS rat model (27). It has also been reported that human PCOS patients had lower BAT activity (28). BAT transplantation significantly increased insulin sensitivity, and ameliorated hyperandrogenism, acyclicity, and infertility in rat and mouse models of PCOS (27, 29). Treatment with rutin, a novel compound that activates BAT, dramatically increased BAT activity, improved systemic insulin resistance, and restored ovarian function (30). These results, at least in part, indicate that increasing amounts of BAT and/or activating endogenous BAT could be a novel therapeutic strategy for PCOS.

Cold exposure is a powerful physiological stimulus for endogenous BAT activation (24, 31). In clinical trials, cold treatment has improved oxidative metabolism, and enhanced the absorption of glucose and fatty acids in BAT (24, 32, 33). In the current study, cold treatment was investigated as a new therapeutic strategy for PCOS.

## Materials And Methods

### Animals

Three-week-old Sprague–Dawley rats were purchased from Charles River Laboratory Animal Technology Co. Ltd, Beijing. Five female rats per cage and three male rats per cage were housed in an Office of Laboratory Animal Welfare-certified animal facility with a 12-h light/dark cycle, and fed rodent chow *ad libitum*. All rat studies were conducted with the approval of the Institutional Animal Care and Use Committee of the Institute of Zoology, Chinese Academy of Sciences. Rats in the cold treatment group were placed in individual cages in a thermostatic incubator maintained at 4°C and kept under a 12-h light/dark cycle for 20 days.

### Vaginal Smears and Estrous Cycle Assessment

Vaginal smears were taken at 3:00 p.m. for 8 days, and H&E staining was conducted on day 8. Estrous cycle stages were determined *via* microscopy. Diestrus consists of a predominance of leukocytes and nucleated epithelial cells. Proestrus consists of round, nucleated epithelial cells. Estrus consists of squamous cornified epithelial cells. Metestrus consists of leukocytes and epithelial cells.

### PCOS Model Establishment

DHEA used to establish the PCOS model was purchased from Yaxing Pharmaceutical Co., Ltd, Shanghai. Three-week-old female rats were injected subcutaneously with DHEA (0.6mg kg^-1^) daily for 20 days. Control rats were injected with water under the same administration regimen. Rats without two typical 4-day estrous cycles within eight days were judged as PCOS model rat. After successful induction of the PCOS model, DHEA was continuously injected until sacrifice or fertility assessment experiment.

### Fertility Assessment

Female rats were mated with healthy male rats. Successful mating was determined based on the presence of a vaginal plug. The female rats underwent natural delivery. The number of newborn pups was counted and recorded.

### Blood Analysis

Blood samples were collected from the hearts of rats under Avertin anesthesia. Plasma was separated and stored at –80°C. Enzyme-linked immunosorbent assay kits were used to detect Testosterone (CSB-E05100r, cusabio), Estradiol (CSB-E05110r, cusabio), Luteinizing hormone (CSB-e12654r, cusabio), and Follicle-stimulating hormone (CSB-E06869r, cusabio).

### Histology Analysis

Tissues were fixed in 4% paraformaldehyde for 36 hours at room temperature and then embedded in paraffin. Five-micrometer-thick sections were stained with hematoxylin and eosin, and assessed *via* microscopy (DS-RI1, Nikon). Numbers of corpora lutea and cystic follicles were counted based on morphology.

### Gene Expression Analysis

Total RNA from whole ovary was extracted using a trizol reagent (15596018; Invitrogen). Reverse transcription of total RNA was performed with a high-capacity cDNA reverse transcription kit (R312-01/02, Vazyme). cDNA was diluted to 10 ng uL^-1^. Real-time PCR analysis (ABI Prism VIIA7, Applied Biosystems) was performed with a SYBR Green Master Mix (Q511-AA, Vazyme) and normalized based on cyclophilin expression. The mRNA expression of related genes was normalized against that of *Cyclophilina*. The primers used for real-time PCR are shown in **Table 1**.

**Table 1:** Primers for RT-PCR, related to methods.

Gene	Forward primer	Reverse primer
17β-HSD	TGTGGGTGCTGTACTGGATGTGAA	ACTTGCTGGCACAGTACACTTCGT
StAR	TGTTAAGGACTGCCCACCACATCT	TGTCCTTGGCTGAAGGTGAACAGA
CYP19A1	GGCATGCACGAGAATGGCATCATA	CAGCCTGTCCAAATGCTGCTTGAT
SRD5A1	CGACCTGCCTGGTTCATACA	AAAACCAGCGTCCTTTGCAC
IFNγ	TGTCATCGAATCGCACCTGAT	CACCGACTCCTTTTCCGCT
IL18	CCACTTTGGCAGACTTCACTG	GTCTGGGATTCGTTGGCTGTT
CCL2	TCCACCACTATGCAGGTCTCT	GTGGGGCATTAACTGCATCTGG
CCL20	CAGCACTGAGCAGATCAATTCCT	CAGTCAAAGTTGCTTGCTGCTTCT
Cyclophlin	GTCTGCTTCGAGCTGTTTGC	CACCCTGGCACATGAATCCT

### Western Blotting Analysis

Proteins were purified in RIPA lysis buffer containing a protease and phosphatase inhibitor mixture (Roche Diagnostics). Protein concentrations were tested *via* a BCA assay kit (Pierce Diagnostics). Proteins were separated on SDS/PAGE gels, transferred to polyvinylidene difluoride membranes (Millipore), blocked in 5% skim milk (OXOID) in TBST (0.02 M Tris base, 0.1% Tween 20, 0.14 M NaCl pH 7.4), and incubated with primary antibodies overnight at 4°C. The primary antibodies used were anti-uncoupling protein 1 (UCP1) (ab209483, Abcam) and anti-HSP90 (4874, Cell Signaling Technology). Goat anti-Rabbit IgG H&L (A0277, Beyotime). The primary antibody was diluted at a ratio 1:1000; The secondary antibody was diluted at a ratio 1:5000. Signals were detected with super signal west pico chemiluminescent substrate (Pierce). Intensity values of the bands were analyzed *via* ImageJ software (National Institutes of Health, Bethesda, MD, USA).

### Statistical Analysis

Comparisons between groups were assessed *via* one-way analysis of variance with Tukey’s post-hoc test, or Student’s *t* tests. Statistical significance was set at *p* < 0.05.

## Results

### Effects of Cold Treatment on BAT Activation

**Figure 1:**
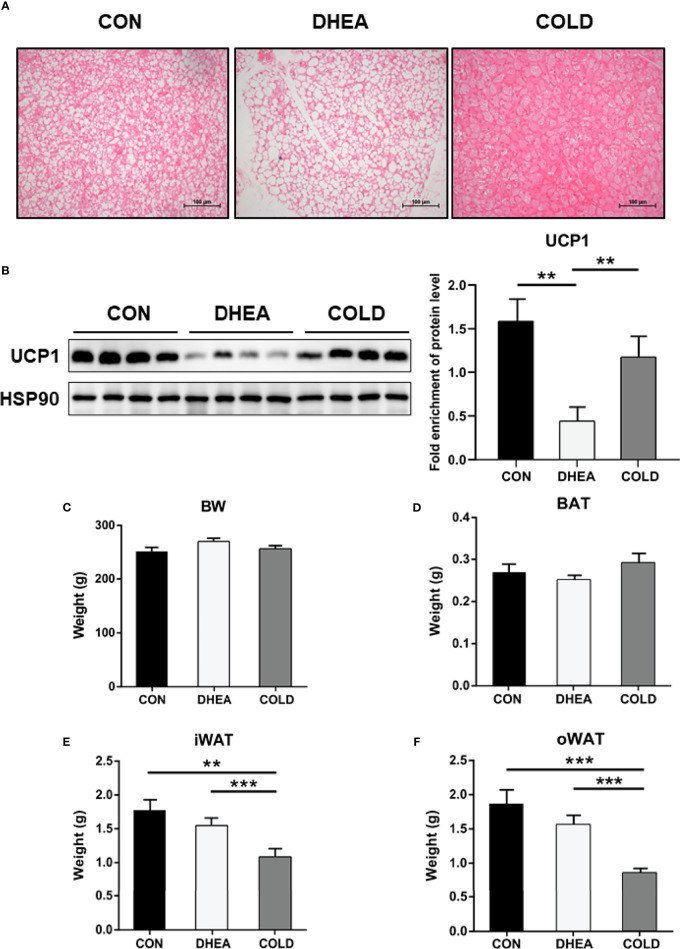
Cold treatment improved activity of BAT in PCOS rat. **(A)** H&E staining of BAT. Scale bar: 100 μm. **(B)** Protein expression of UCP1 in BAT. The weight of body **(C)**, BAT **(D)**, iWAT **(E)** and oWAT **(F)**. Data are means ± SEM. **(A)** n = 3/group; **(B)** n = 4/group; **(C–F)** CON and DHEA treatment (n = 10/group), and COLD treatment (12 = 10/group). One-way ANOVA with Tukey post-hoc test was used to compare groups. **P < 0.01, ***P < 0.001.

BAT whitening is one of the most obvious phenotypes in the PCOS rat model. Increased adipocyte size identified *via* histological analysis was consistent with the reduction of multiple small lipid droplets in brown adipocytes of PCOS rats, indicating that DHEA triggered brown adipocyte hypertrophy. After cold treatment, DHEA-induced BAT hypertrophy was significantly reversed. These results suggest that BAT was effectively activated by cold treatment (**Figure 1A**). BAT generates heat by uncoupling of mitochondrial ATP synthesis which is primarily achieved by UCP1 (34). UCP1 expression was decreased in the DHEA group, and restored to a normal control level after cold treatment (**Figure 1B**). Cold treatment had no effect on body weight or BAT weight (**Figures 1C, D**). Inguinal subcutaneous white adipose tissue (iWAT) and visceral WAT around ovary (oWAT) were significantly reduced by cold exposure (**Figures 1E, F**). Collectively, these results suggest that cold treatment activated BAT and enhanced fat consumption.

### Effects of Cold Treatment on Hyperandrogenism and Ovarian Acyclicity

**Figure 2:**
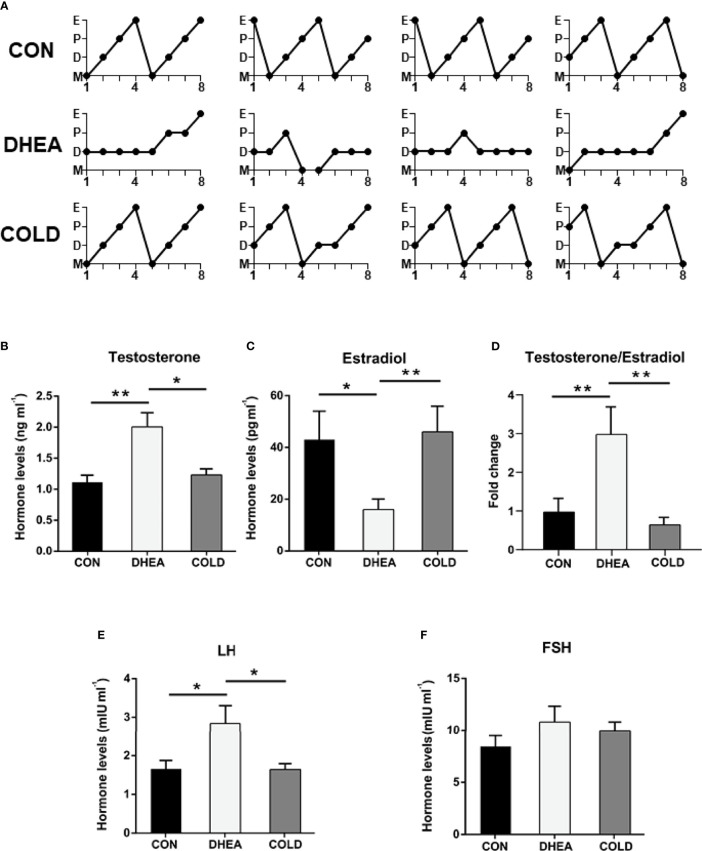
Cold treatment ameliorated hyperandrogenism and ovarian acyclicity in PCOS rat. **(A)** Representative estrous cycles. The hormone levels of testosterone **(B)**, estradiol **(C)**, the normalized testosterone/estradiol ratio **(D)**, LH **(E)** and FSH **(F)**. Data are means ± SEM. **(B–E)** CON and COLD treatment (n = 8/group), and DHEA treatment (n = 7/group). One-way ANOVA with Tukey post-hoc test was used to compare groups. *P < 0.05, **P < 0.01.

Abnormal menstruation is a typical symptom in PCOS. The normalization of menstruation is an important aim of PCOS treatment. Therefore, we investigated the menstrual cycle after 20 days of cold treatment. Normal menstruation was observed in 8/12 PCOS rats after cold treatment, and in 3/10 rats in the DHEA group (**Figure 2A** and **Table 2**). Hyperandrogenemia and abnormally low estradiol were significantly recovered to normal control levels after cold treatment (**Figures 2B, C**). The testosterone/estradiol ratio is an important parameter for the diagnosis of PCOS which was significantly increased in PCOS rats and significantly decreased to the control level after cold treatment (**Figure 2D**). There were no significant differences in follicle-stimulating hormone (FSH), but the abnormally increased luteinizing hormone (LH) level in PCOS rat plasma was significantly decreased after cold treatment (**Figures 2E, F**). Collectively, these results indicate that cold treatment can restore ovarian cyclicity and reverse hyperandrogenism.

**Table 2:** Summary of the estrous cycles.

Group	Total no.	Normal estrous cycle	Abnormal estrous cycle
CON	10	10	0
DHEA	10	2	8
COLD	12	8	4

### Effects of Cold Treatment on DHEA-induced Ovarian Dysfunction

**Figure 3:**
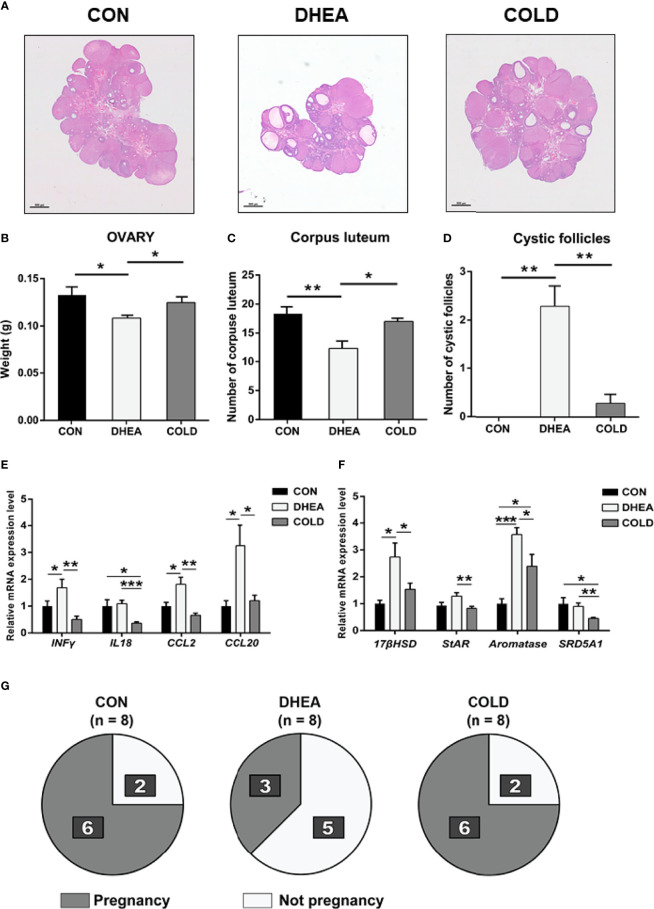
Cold treatment improved ovary dysfunction and normalized fertility in PCOS rat. **(A)** Hematoxylin and eosin staining of representative ovaries. Scale bar: 800 μm. **(B)** The weight of ovary. **(C)** The number of corpus luteum. **(D)** The number of cystic follicles. **(E)** The mRNA level of inflammation-related genes in ovary. **(F)** The mRNA level of ovarian steroidogenic enzymes. **(G)**The number of pregnancy rats and not pregnancy rats are shown in dark and bright. Data are means ± SEM. **(A, C, D)** CON and DHEA treatment (n = 8/group), and Cold treatment (n = 10/group); **(B, E, F)** Con and DHEA treatment (n = 10/group), and Cold treatment (n = 10/group). **(G)** CON, DHEA treatment and COLD treatment (n= 8/group). One-way ANOVA with Tukey post-hoc test was used to compare groups. *P < 0.05, **P < 0.01, ***P < 0.001.

Compared with the normal control group, the ovaries in the DHEA group exhibited typical PCOS characteristics with excessive cystic follicles and an absence of corpus luteum. In the DHEA group, there were abnormal expression levels of ovarian steroidogenic enzymes and ovarian inflammation. After cold treatment, there was a significant reduction in the number of cystic follicles. In histopathological analysis, the number of corpus luteum was significantly increased after cold treatment (**Figures 3A–D**). Cold treatment ameliorated or reduced abnormal expression of ovarian steroidogenic enzymes such as *17-β hydroxysteroid dehydrogenase* (*17β-HSD*), *steroidogenic acute regulatory protein* (*StAR*), *cytochrome P450, family 19 subfamily a polypeptide 1* (*CYP19A1*), and *steroid 5 alpha-reductase 1* (*SRD5A1*) in the DHEA-induced PCOS rat model (**Figure 3E**). It also significantly reversed the expression of inflammation factors such as *interferon gamma* (*IFNγ*), *interleukin 18*(*IL18*), *C-C motif chemokine ligand 2* (*CCL2*), and *C-C motif chemokine ligand 20* (*CCL20*) to normal control levels (**Figure 3F**). These results indicated that cold treatment has positive effects on ovarian dysfunction. As well as the recovery of acyclicity and improvement of hyperandrogenism and ovarian dysfunction, we investigated whether cold treatment could normalize fertility in PCOS rats. Female rats in three groups were mated with healthy male rats and the numbers of successful pregnancies and deliveries were counted. The proportion of pregnancies in the cold treatment group was 6/8, which was significantly higher than the 3/8 in the DHEA group. Deliveries were normal (pup numbers ranged from 1–16) and there was no significant difference between the DHEA group and the cold treatment group (**Figure 3G**, **Table 3**). Collectively, these results indicated that cold treatment had obvious positive effects on infertility in PCOS rats.

**Table 3:** Fertility assessment.

Group0	Rat number(n=8)	copulatory plugs	Litter size	Average litter size
CON	9191	Yes	15	
	9196	Yes	18	
	9107	Yes	16	
	6169	Yes	13	15
	9578	Yes	14	
	9108	Yes	15	
	6318	Yes	0	
	6319	No	0	
DHEA	8551	Yes	13	
	6123	No	0	13
	8553	Yes	0	
	6179	Yes	0	
	8554	Yes	14	
	6119	Yes	12	
	8556	Yes	0	
	6178	Yes	0	
COLD	6162	Yes	14	
	6163	No	0	
	7141	Yes	11	
	6176	Yes	16	
	6177	Yes	0	12
	6120	Yes	1	
	6164	Yes	15	
	6121	Yes	15	

## Discussion

BAT has a marked thermogenic capacity to increase energy expenditure and is thus recognized as a promising target for the treatment of obesity and other metabolic syndromes. In previous studies, BAT activity was reduced in PCOS women and PCOS model rats (27, 28). In multiple studies, improvement of PCOS was accompanied by recovery of BAT activity (30, 35). Research also indicates that BAT transplantation can reverse polycystic ovaries, insulin resistance, and infertility in PCOS rats and mice (27, 29). Notably, BAT transplantation is a technique that requires a high level of clinical complexity, which increases the challenges of its clinical application. Our group previously demonstrated that the small molecule rutin, a BAT activator, significantly improved systemic insulin resistance and restored ovarian function in PCOS rats (30). However, it would take a long time for rutin to be approved for PCOS clinical treatment. Therefore, it is necessary to investigate additional therapies for PCOS.

Cold exposure is a classic and effective strategy for BAT activation. Under low ambient temperature, BAT responds to sympathetic nervous system signals and efficiently converts the chemical energy stored in lipid into heat energy, which helps the body adapt to environmental challenge. Moreover, cold-induced thermogenesis in BAT also might be a promising therapeutic effect for the treatment of metabolic diseases. In a clinical study, 4 weeks of cold exposure (10°C, 2 hours) elicited a 45% increase in BAT volume and a 2-fold increase in total BAT oxidative metabolism (33). In another study, daily cold exposure (17°C, 2 hours) for 6 weeks resulted in increased BAT activity, cold-induced increments of energy expenditure, and a concomitant decrease in body fat mass (24). In the present study, the therapeutic effects of cold treatment were investigated in PCOS rats. To our knowledge, it is the first time to apply cold exposure into PCOS treatment. The results indicated that addressing the functional abnormalities of adipose tissue is necessary for the treatment of reproductive dysfunction.

In the current study, BAT activity was restored to normal control levels after cold treatment as evidenced by increased numbers of adipocytes with multilocular lipid droplets, and restoration of UCP1 expression. In addition, 8/12 PCOS rats exhibited normal menstruation in the cold treatment group, whereas only 2/10 PCOS rats exhibited normal menstruation in the DHEA group. These results indicated that cold treatment could effectively reverse acyclicity. Cold treatment also had positive effects on hyperandrogenemia. DHEA-induced abnormally high testosterone and luteinizing hormone recovered to normal levels after cold treatment, and cold treatment significantly reduced the expression of steroidogenic enzymes as well as inflammatory factors in the ovaries of PCOS rats. Histological investigations indicated that cold treatment could significantly increase corpus luteum numbers and reduce cystic follicle numbers, indicating that ovulation was recovered to a normal level. Concordant with these results, the successful pregnancy rate in the cold treatment group of 6/8 was twice that in the DHEA group (3/8), indicating that cold treatment could improve fertility in PCOS rats.

It is unclear how cold treatment improves PCOS. BAT secretes batokines that regulate whole-body energy homeostasis (26, 36). Fibroblast growth factor 21 (FGF21) is a pleiotropic protein involved in lipid and glucose metabolism, and energy homeostasis (37). Cold exposure reportedly significantly increased FGF21 expression in BAT (33). Neuregulin 4 (Nrg4), another brown fat-enriched secreted factor, protects against diet-induced insulin resistance and hepatic steatosis (38). It has also been shown that BAT secretes adiponectin which stimulates fatty acid oxidation, inhibits gluconeogenesis, enhances insulin sensitivity, reduces inflammation, and has anti-apoptotic properties (39). In some previous studies, human PCOS patients and PCOS model rats exhibited significantly lower adiponectin values. In the current study, administration of adiponectin could significantly improve DHEA-induced PCOS in rats (40, 41). Whether batokines are involved in PCOS pathophysiology is unknown, and this important question warrants further investigation.

In conclusion, the results of the present study suggest that the development of PCOS is closely associated with endogenous reduced BAT activity. Cold treatment was identified as a potential new therapeutic strategy for PCOS, and further study is required to investigate the clinical application of this strategy.

## Data Availability Statement

The original contributions presented in the study are included in the article/**Supplementary Material**. Further inquiries can be directed to the corresponding authors.

## Ethics Statement

The animal study was reviewed and approved by the Institutional Animal Care and Use Committee of the Institute of Zoology, Chinese Academy of Sciences.

## Author Contributions

Conceived and designed the experiments: RCY, CLY, HQZ, and WZJ. Performed the experiments: RCY, CLY, HQZ, MD, HLZ, XXJ, and KXZ. Analyzed the data: RCY, CLY, and QLZ. Wrote the paper: RCY and HQZ. Edited the manuscript: LC, RJ, ZYC, SLY, and YYH. All authors contributed to the article and approved the submitted version.

## Funding

This study was supported by the National Key Research and Development Program of China (grant number 2017YFC1001003), the Strategic Collaborative Research Program of the Ferring Institute of Reproductive Medicine (grant number FIRMC180304), the National Natural Science Foundation of China (grant number 81770834), the National Natural Science Foundation of China (grant number 81770577).

## Conflict of Interest

The authors declare that the research was conducted in the absence of any commercial or financial relationships that could be construed as a potential conflict of interest.

## Publisher’s Note

All claims expressed in this article are solely those of the authors and do not necessarily represent those of their affiliated organizations, or those of the publisher, the editors and the reviewers. Any product that may be evaluated in this article, or claim that may be made by its manufacturer, is not guaranteed or endorsed by the publisher.
