# Supplementary figures and images for: Brown Adipose Tissue Activation by Cold Treatment Ameliorates Polycystic Ovary Syndrome in Rat
Source: Front Endocrinol (Lausanne). 2021 Oct 14;12:744628. doi: 10.3389/fendo.2021.744628 (PMC8552032; doi:10.3389/fendo.2021.744628)

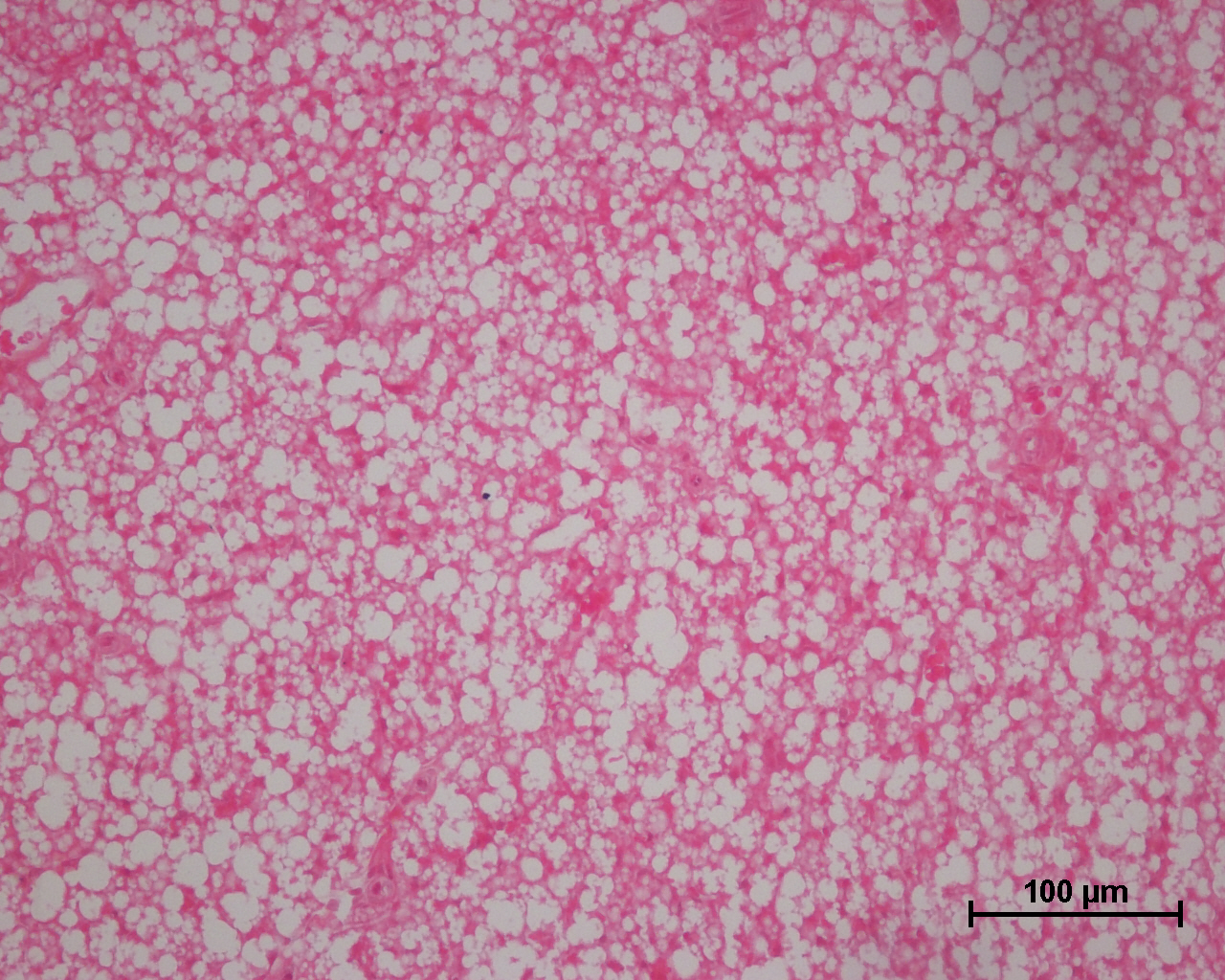

Supplement: Supplementary file 2 [file Image_1.jpeg]

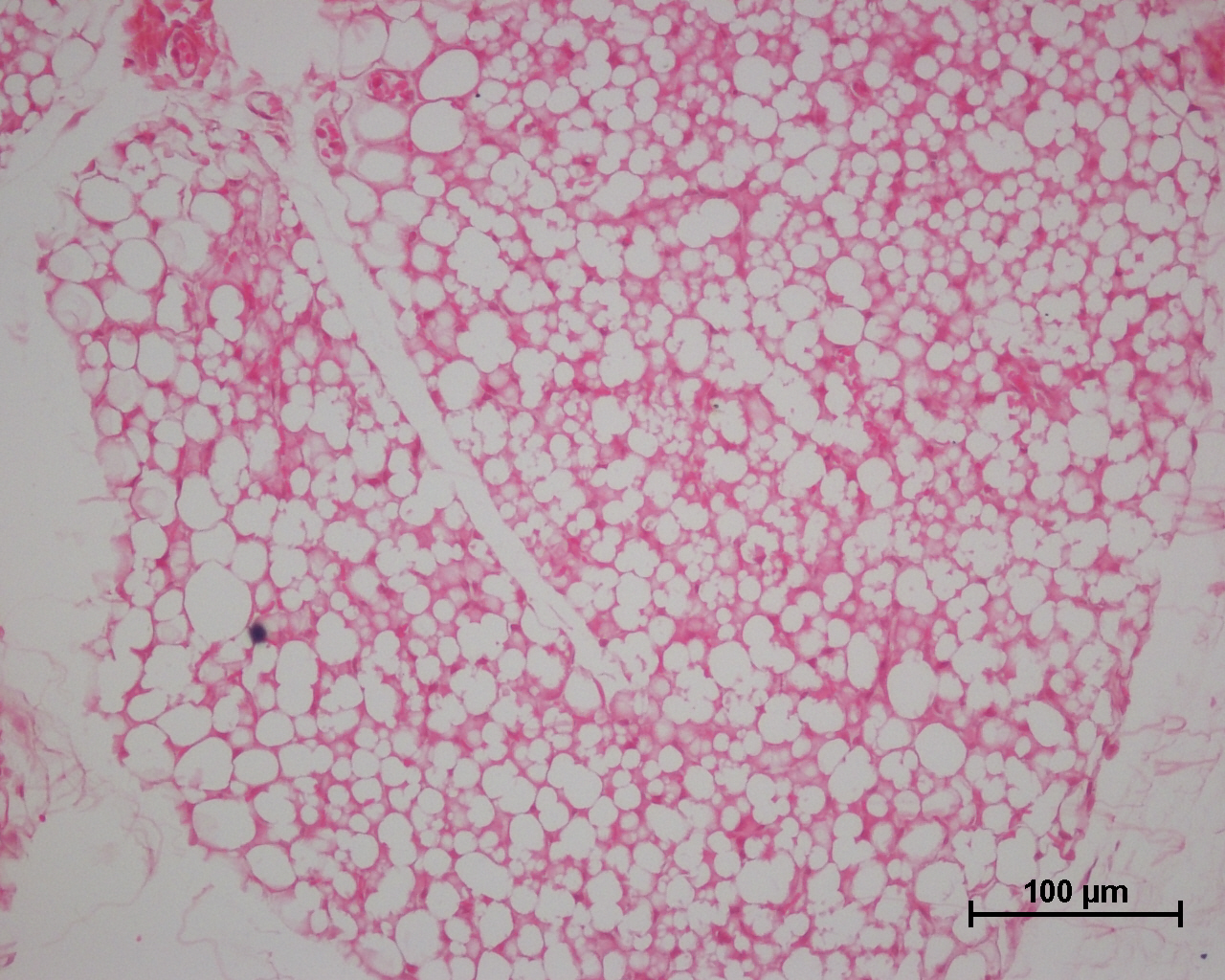

Supplement: Supplementary file 3 [file Image_2.jpeg]

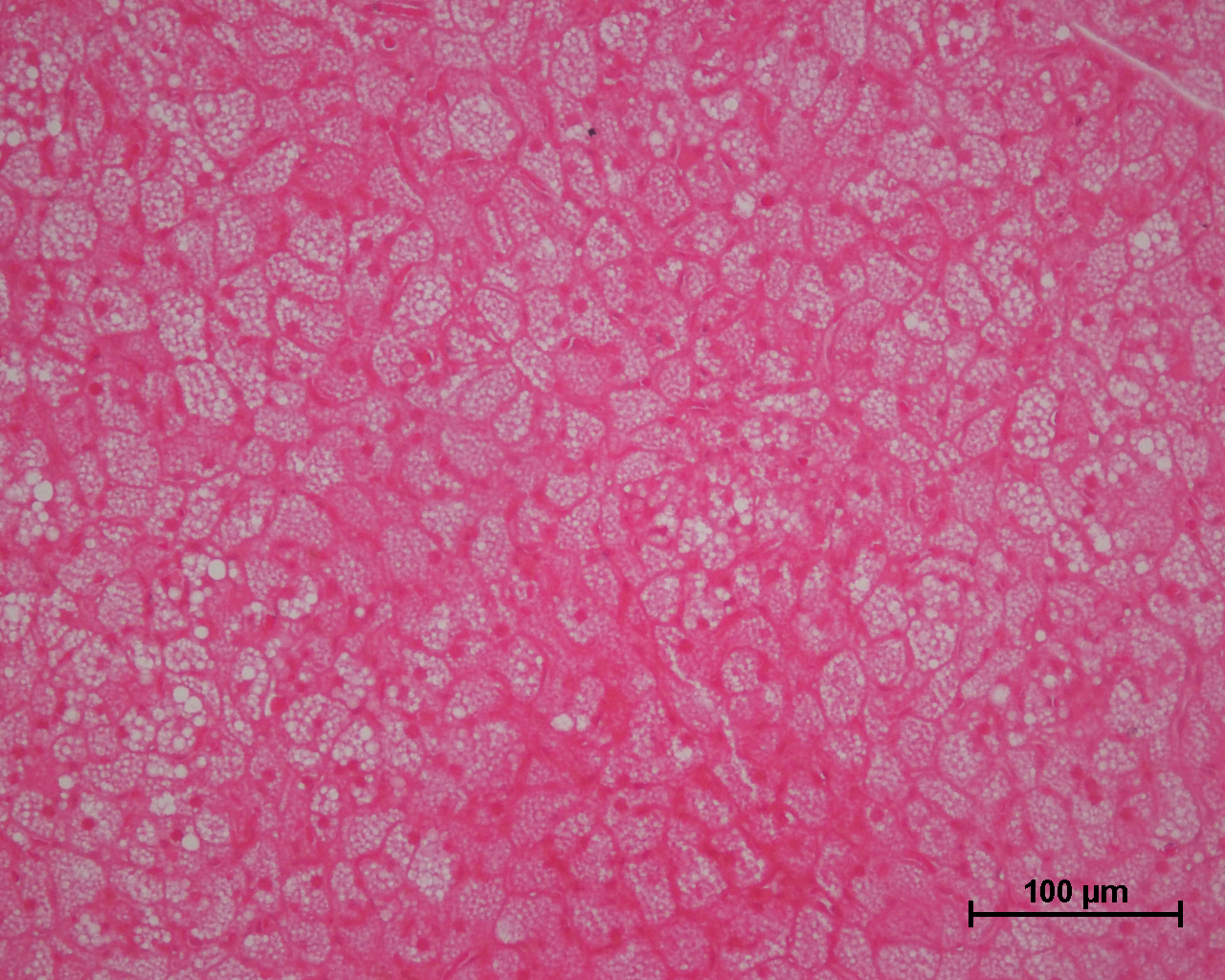

Supplement: Supplementary file 4 [file Image_3.jpeg]

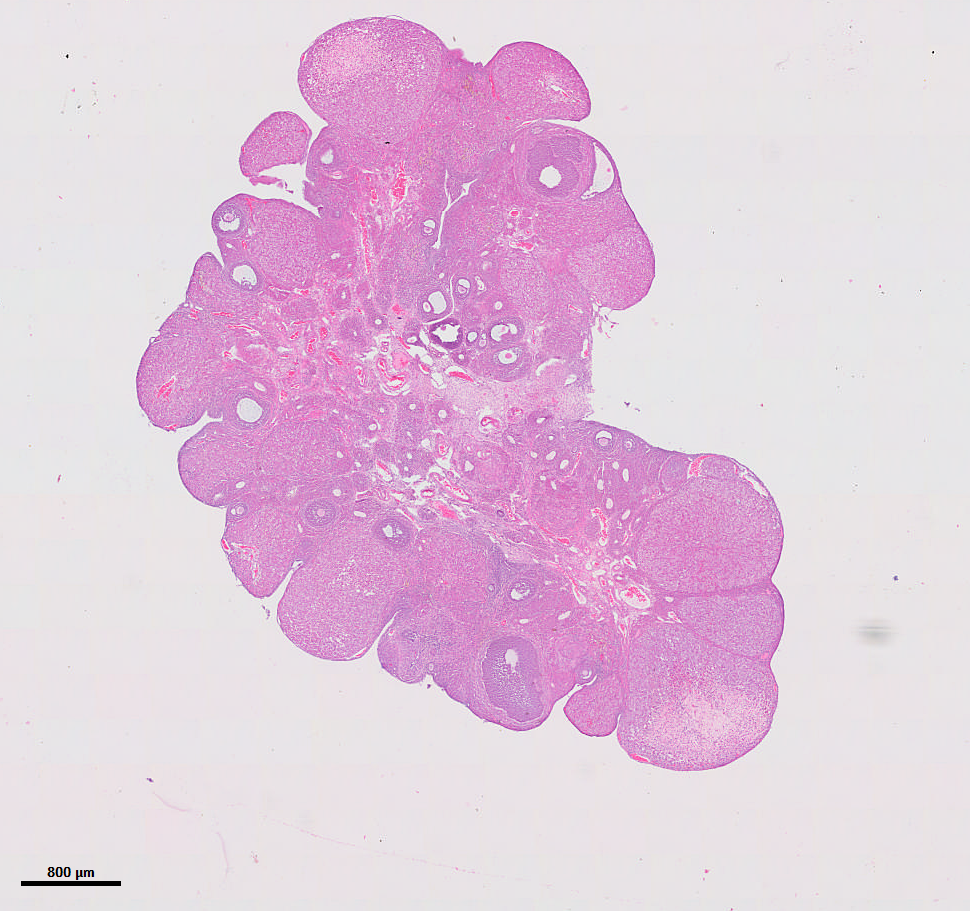

Supplement: Supplementary file 5 [file Image_4.tif]

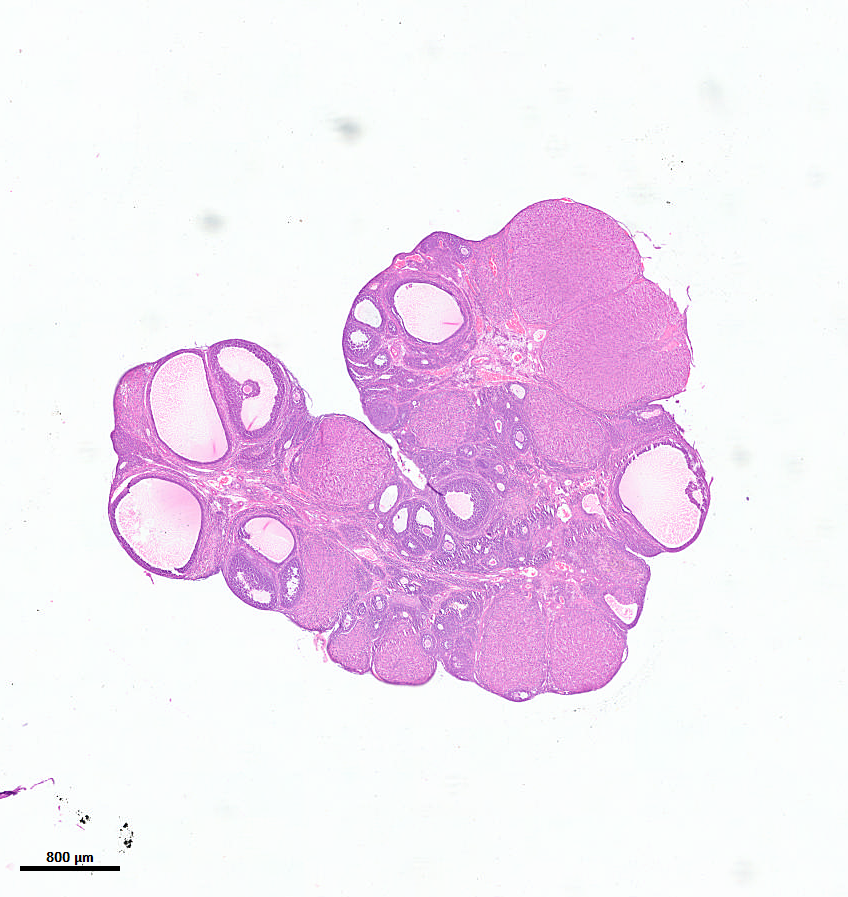

Supplement: Supplementary file 6 [file Image_5.tif]

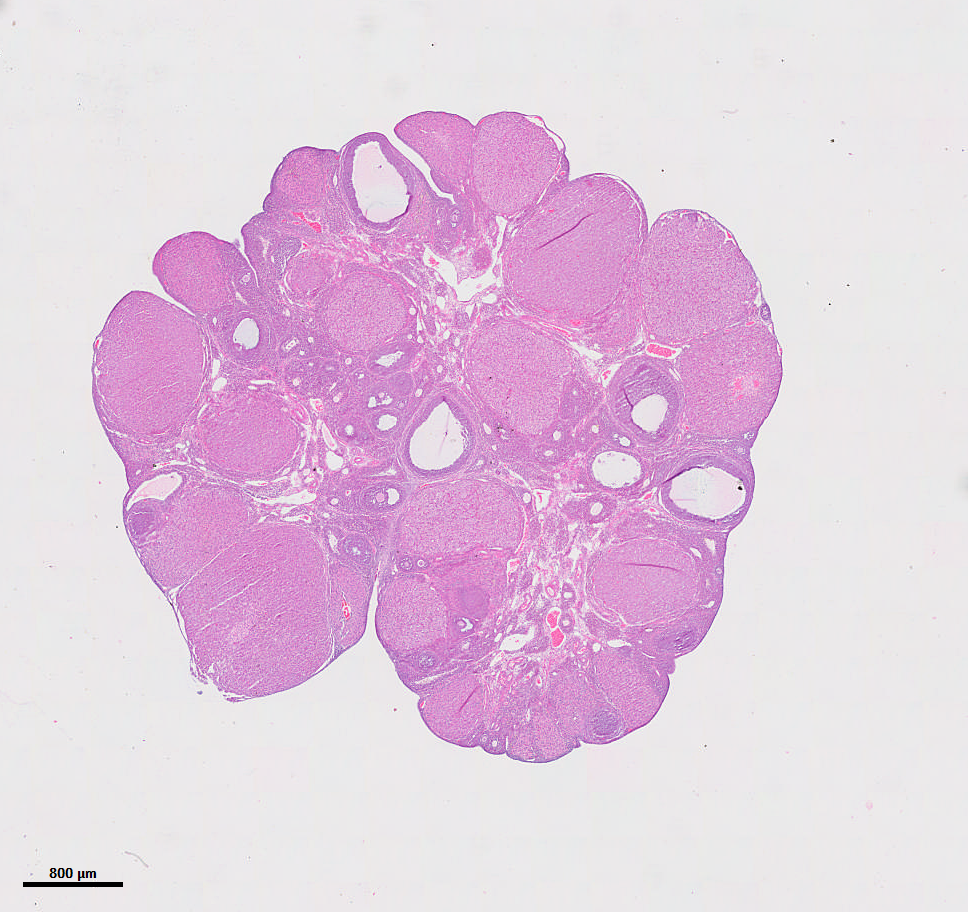

Supplement: Supplementary file 7 [file Image_6.tif]

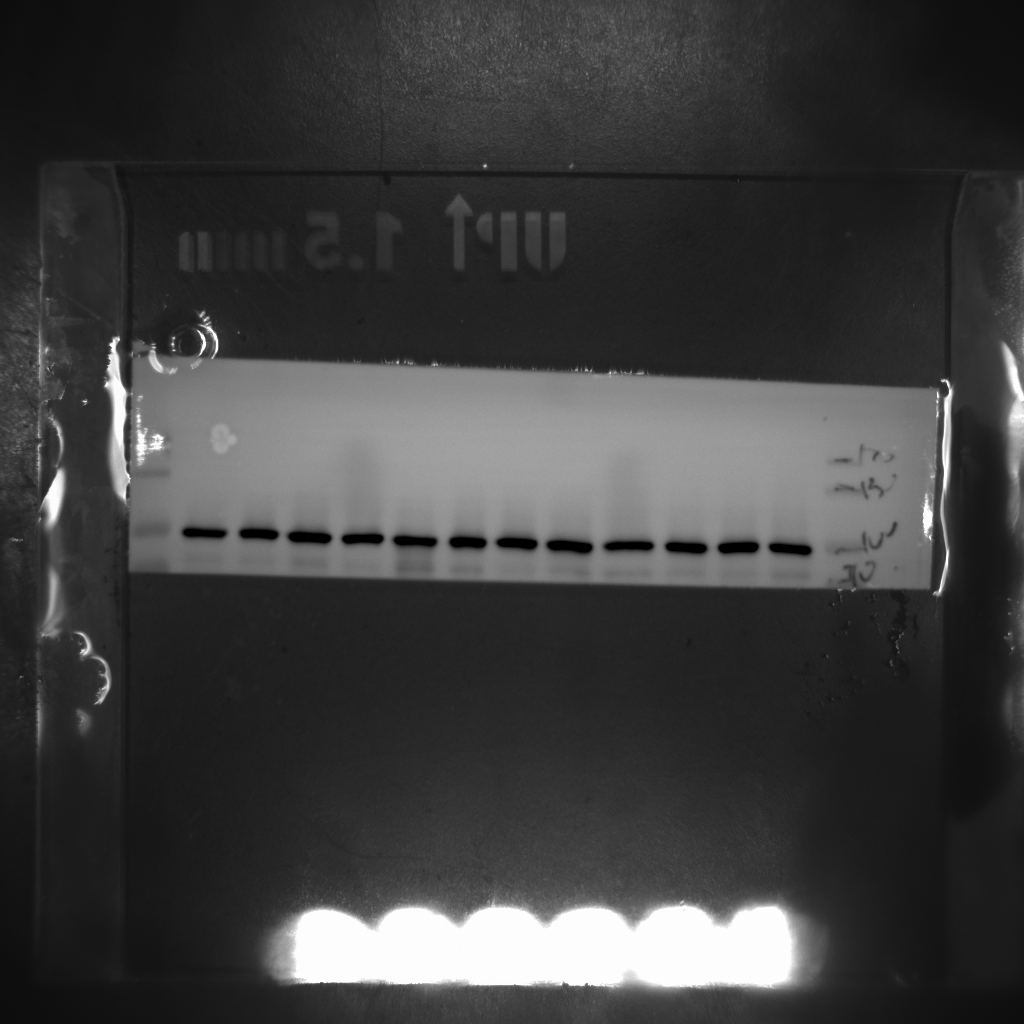

Supplement: Supplementary file 8 [file Image_7.tif]

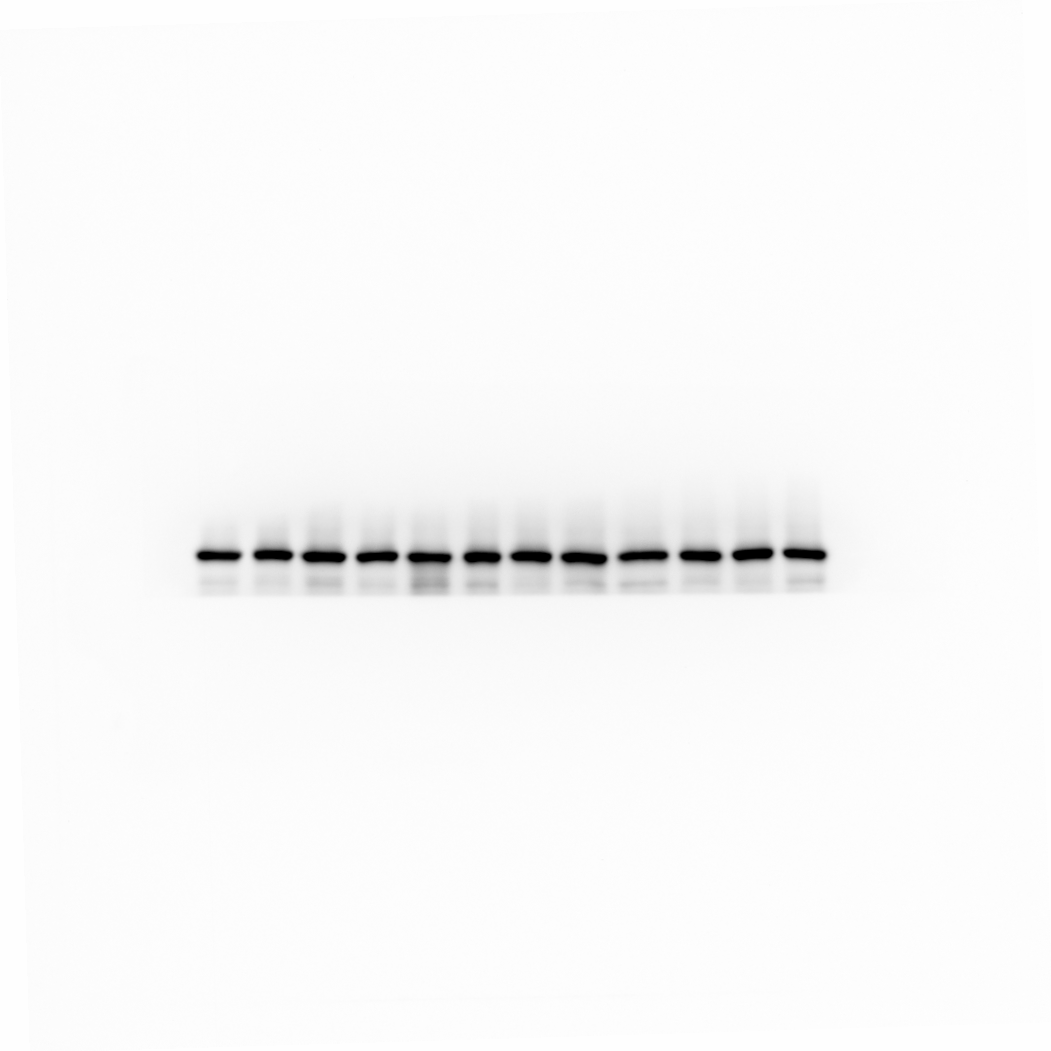

Supplement: Supplementary file 9 [file Image_8.tif]

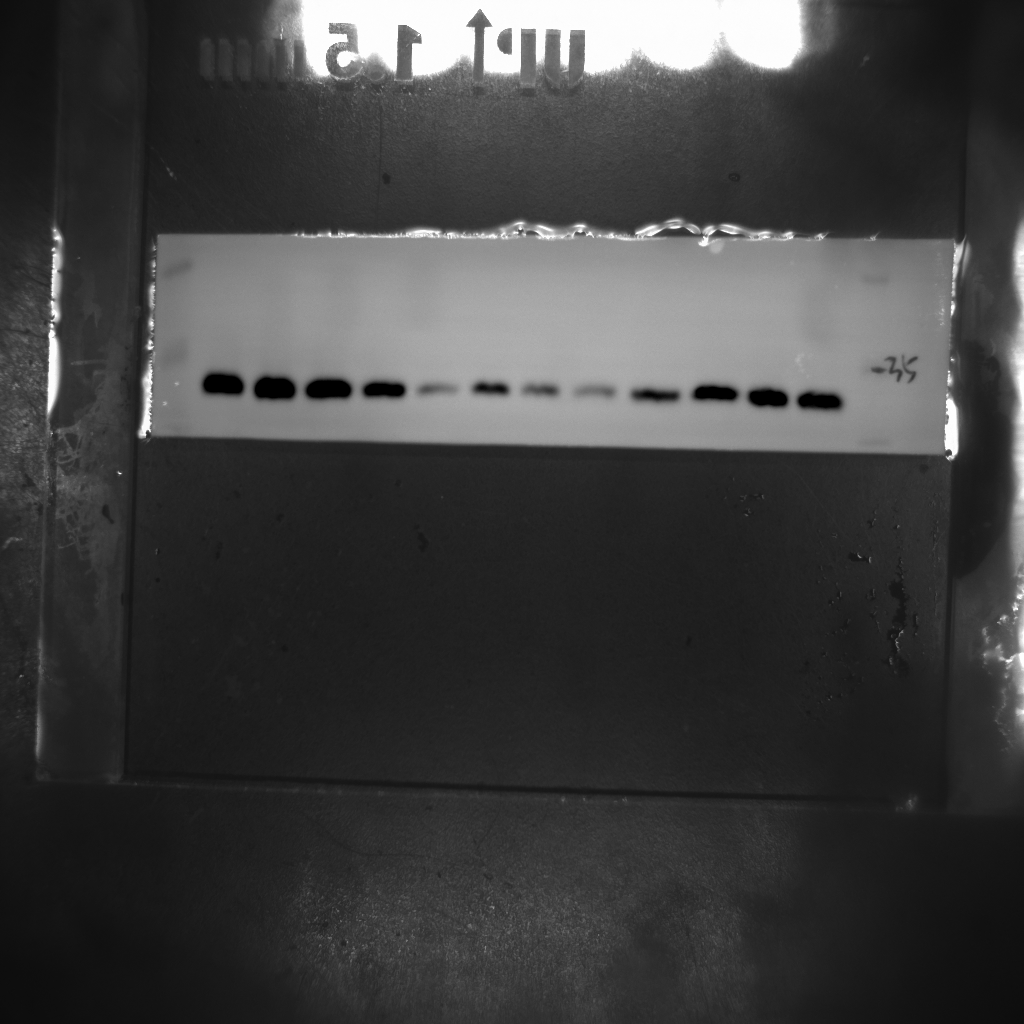

Supplement: Supplementary file 10 [file Image_9.tif]

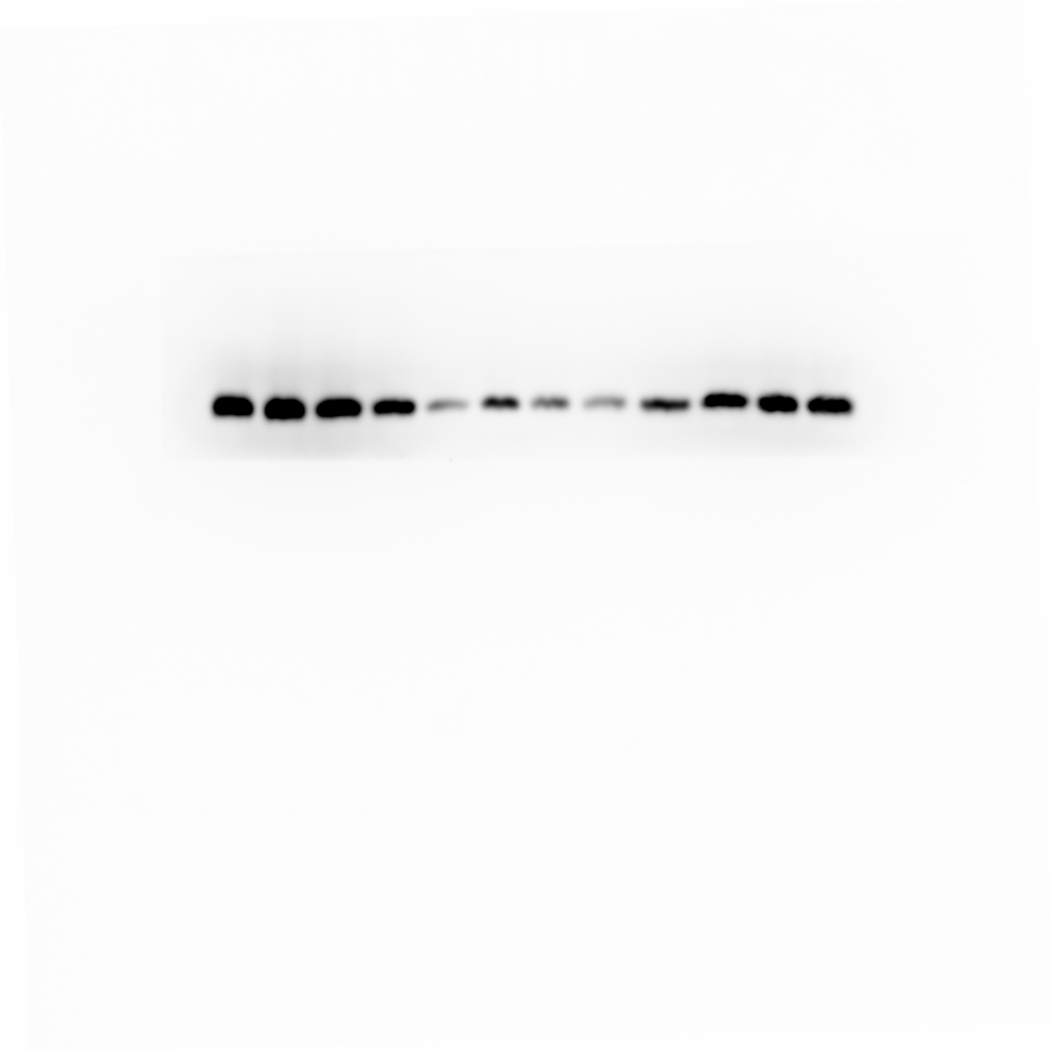

Supplement: Supplementary file 11 [file Image_10.tif]
